# Preparedness of the Ghana Health Service for field epidemiology and applied biostatistics: a systematic review protocol of infectious disease surveillance, outbreak investigation methodologies, and statistical modeling capacities in resource-limited settings

**DOI:** 10.3389/fpubh.2026.1803063

**Published:** 2026-06-12

**Authors:** Victor Luckyboy Dzramado, William Wilberforce Amoah, Samuel Antwi, Joana Edem Koto, Doris Hagan

**Affiliations:** 1 Cape Coast Teaching Hospital, Cape Coast, Ghana; 2 Department of Emergency and Critical Care Nursing, School of Nursing and Midwifery, Kwame Nkrumah University of Science and Technology, Kumasi, Ghana; 3 Department of Epidemiology and Disease Control, School of Public Health, University of Ghana, Legon, Accra, Ghana; 4 Kintampo Health Research Centre, Kintampo, Ghana; 5 Department of Public Health Nursing, School of Nursing and Midwifery, Kwame Nkrumah University of Science and Technology, Kumasi, Ghana

**Keywords:** biostatistics, disease surveillance, epidemic preparedness, field epidemiology, Ghana Health Service, health systems strengthening, outbreak investigation, public health capacity

## Abstract

**Background:**

Infectious disease outbreaks pose significant threats to global health security, with resource-limited settings in West Africa bearing a disproportionate burden. Despite sustained investments in field epidemiology training and surveillance system strengthening, no comprehensive systematic synthesis exists of Ghana Health Service preparedness for field epidemiology and applied biostatistics. This protocol addresses the primary research question: What is the current level of preparedness of the Ghana Health Service for field epidemiology and applied biostatistics, as assessed across World Health Organization International Health Regulations core capacity domains? Preparedness is operationally defined as the measurable capacity of the Ghana Health Service to detect, investigate, confirm, and respond to infectious disease events across the eight WHO IHR core capacity domains, including surveillance, human resources, laboratory systems, and response mechanisms.

**Methods and analysis:**

This systematic review follows PRISMA 2020 guidelines and is registered with PROSPERO (CRD420261299788). Searches will be conducted in PubMed/MEDLINE, African Index Medicus, African Journals Online, and grey literature sources for studies published from January 2010 to present. Eligible studies include those describing field epidemiology capacity, surveillance system performance, outbreak investigation preparedness, biostatistical capacity, training program outcomes, infrastructure, or health workforce within the Ghana Health Service. Two independent reviewers will screen citations, extract data, and assess quality using study-design-appropriate tools including the JBI Critical Appraisal Checklist, CASP Qualitative Checklist, and Mixed Methods Appraisal Tool. Primary outcomes are overall field epidemiology preparedness level measured using Joint External Evaluation and State Party Annual Reporting scores, and surveillance system performance with outbreak response capacity. Secondary outcomes include field epidemiology workforce capacity, statistical modeling and biostatistical capacity, and infrastructure and governance systems. Narrative synthesis is the primary analytic approach. Meta-analysis will be conducted where sufficient comparable studies with acceptable methodological homogeneity are identified.

**Discussion:**

This review will provide the first comprehensive assessment of Ghana Health Service field epidemiology preparedness mapped against WHO IHR core capacities, generating actionable evidence-based recommendations applicable to similar resource-limited settings across Africa.

**Ethics and dissemination:**

No ethical approval is required. Results will be disseminated through peer-reviewed publication, conference presentations, and policy briefs for the Ghana Health Service, Ministry of Health, and international stakeholders.

**Systematic review registration:**

https://www.crd.york.ac.uk/PROSPERO/view/CRD420261299788, identifier CRD420261299788.

## 1 Introduction

### 1.1 Global context and rationale

Infectious disease outbreaks continue to pose significant threats to global health security, with devastating social, economic, and health consequences. Globally, the burden of epidemic-prone diseases falls disproportionately on low- and middle-income countries (LMICs), where health system capacity to detect and respond to outbreaks remains inadequate (1, 2). The World Health Organization (WHO) International Health Regulations (IHR) 2005 mandate that all member states develop and maintain core capacities for disease surveillance, outbreak detection, and rapid response (1). Yet substantial gaps persist globally, with LMICs facing particular challenges in achieving IHR compliance due to resource constraints, workforce limitations, and infrastructure deficits (2, 3).

Recurrent epidemic events including the West African Ebola virus disease outbreak (2014 to 2016), cholera outbreaks, cerebrospinal meningitis epidemics, and the COVID-19 pandemic (2020 to present) have highlighted critical failures in preparedness and response capacity, particularly in sub-Saharan Africa (4–6). These events demonstrated that effective disease surveillance and rapid outbreak response require not only robust infrastructure but also a competent workforce trained in field epidemiology and applied biostatistics (7, 8). In response, countries have established Field Epidemiology Training Programs (FETPs) to build workforce capacity. The Training Programs in Epidemiology and Public Health Interventions Network (TEPHINET) now coordinates FETPs in over 80 countries globally (9, 47, 49, 54, 55). The African Field Epidemiology Network (AFENET) provides regional coordination and technical support across the continent, contributing measurably to improved outbreak detection and response (10, 11, 44). However, challenges in graduate retention, deployment, and sustainable funding continue to limit impact in many settings (12).

Field epidemiology, defined as the application of epidemiological methods to investigate and control health problems in defined populations, forms the foundation of effective outbreak detection and response (13). Applied biostatistics and statistical modeling are equally critical for analyzing surveillance data, detecting trends and anomalies, forecasting epidemic trajectories, and informing evidence-based policy decisions (8, 14, 15, 45, 46). Despite the recognized importance of these capacities, rigorous synthesis of evidence on the preparedness of national health services in resource-limited settings remains limited, particularly in West Africa (7, 16).

### 1.2 Ghana country context and health system

Ghana is a lower-middle-income country in West Africa with a population of approximately 33 million people (17). The country operates a decentralized health system under the leadership of the Ministry of Health, with the Ghana Health Service (GHS) serving as the principal implementer of public health services across 16 administrative regions (18, 50). The GHS bears primary responsibility for disease surveillance, epidemic intelligence, outbreak investigation, health information management, and laboratory services coordination (19).

Ghana has implemented the WHO-supported Integrated Disease Surveillance and Response (IDSR) strategy since 2003, operating through the District Health Information Management System 2 (DHIMS2) electronic platform (20, 21, 48, 52). Established in 2007, the Ghana Field Epidemiology and Laboratory Training Programme (GFELTP) trains field epidemiologists through a two-year advanced residential program, a three-month frontline program, and short-term courses (22, 23, 49). Recent evidence indicates that GFELTP graduates have contributed to surveillance system strengthening and outbreak response, though workforce density, geographic distribution, and sustained retention remain areas of concern (24–26).

Ghana carries a significant burden of epidemic-prone and emerging infectious diseases including malaria, cholera, cerebrospinal meningitis, yellow fever, viral hemorrhagic fevers, zoonotic diseases, and vaccine-preventable diseases (27–29, 51, 53). Recent assessments have identified persistent gaps in the specimen referral system, laboratory diagnostic integration, and epidemiological modeling capacity that continue to limit outbreak detection and response efficiency (8, 30). These gaps are compounded by geographic disparities in health system capacity, particularly between northern and southern regions (21, 31).

For the purposes of this review, preparedness is operationally defined as the measurable capacity of the Ghana Health Service to detect, investigate, confirm, and respond to infectious disease events, assessed across the eight WHO IHR core capacity domains: (i) national legislation and financing; (ii) coordination and national focal point communication; (iii) surveillance; (iv) response; (v) preparedness; (vi) risk communication; (vii) human resources; and (viii) laboratory capacity (1). This framework provides internationally standardized benchmarks against which Ghana’s current status can be systematically measured and compared.

### 1.3 Knowledge gap and study justification

Despite investments in field epidemiology training, surveillance system strengthening, and laboratory capacity building over the past 15 years, comprehensive assessment of Ghana Health Service preparedness remains fragmented across grey literature, published surveillance evaluations, outbreak reports, training program assessments, and academic dissertations (25, 31, 32). No systematic review has synthesized this evidence to provide an integrated picture of preparedness across surveillance, outbreak investigation, workforce, and statistical modeling domains, or to identify specific gaps relative to WHO IHR core capacity standards (16, 33, 34).

The fragmented nature of available evidence makes it difficult for policymakers to identify specific capacity gaps, prioritize interventions, allocate resources efficiently, or benchmark progress against international standards (35, 36). This systematic review addresses these gaps by synthesizing all available evidence on GHS preparedness, mapping findings against the WHO IHR core capacity framework, and providing evidence-based recommendations for capacity strengthening.

#### 1.3.1 Primary research question

What is the current level of preparedness of the Ghana Health Service for field epidemiology and applied biostatistics, as assessed across WHO IHR core capacity domains for infectious disease surveillance, outbreak investigation, and statistical modeling?

### 1.4 Review objectives

#### 1.4.1 Primary objectives

1 To determine the current state of preparedness of the Ghana Health Service for infectious disease surveillance and outbreak investigation, measured against WHO IHR core capacity indicators.
2 To assess the statistical modeling and biostatistical capacities within the Ghana Health Service, defined by specific indicators including biostatistician density, statistical software utilization, use of quantitative models for outbreak forecasting, and evidence-based decision-making processes.

#### 1.4.2 Secondary objectives

3 To evaluate the outcomes of field epidemiology training programs, particularly the GFELTP, in terms of workforce capacity, graduate deployment, and retention.
4 To assess the infrastructure, resources, and systems supporting infectious disease surveillance, outbreak investigation, and statistical analysis.
5 To identify geographic and health system level variations in preparedness and develop evidence-based recommendations for capacity strengthening.

## 2 Methods and analysis

### 2.1 Study design

This protocol describes a systematic review with potential meta-analysis of quantitative, qualitative, and mixed-methods studies examining field epidemiology and biostatistics preparedness of the Ghana Health Service. The review is conducted and reported in accordance with PRISMA 2020 guidelines (37). This protocol has been registered with PROSPERO, registration number CRD420261299788.

The PICOS framework used to define eligibility criteria is summarized in Table 1.

**Table 1:** PICOS framework for the systematic review.

PICOS element	Inclusion criteria	Exclusion criteria
Population	GHS workforce (field epidemiologists, surveillance officers, biostatisticians, laboratory personnel, data managers); GHS systems (IDSR, DHIMS2, GFELTP, outbreak units, reference labs)	Studies focused exclusively on clinical management without surveillance or epidemiological components; studies outside Ghana without comparative Ghana data
Intervention/exposure	GFELTP training (two-year advanced, three-month frontline, short courses); surveillance system interventions (IDSR, DHIMS2, mobile reporting); infrastructure strengthening (laboratory diagnostics, statistical software, GIS); organizational interventions (rapid response teams, SOPs, quality improvement)	Interventions focused exclusively on clinical treatment; vaccination campaigns without surveillance components; administrative reforms unrelated to field epidemiology
Comparator	Temporal (pre/post), international standards (WHO IHR, JEE thresholds, SPAR benchmarks), geographic (cross-regional, urban–rural), intervention-based, or no comparator (descriptive studies)	Comparators from high-income countries without adjustment for contextual differences; outdated or non-validated assessment tools
Outcomes	See Table 2	Outcomes unrelated to preparedness for infectious disease surveillance, outbreak investigation, or biostatistical capacity
Study design	Quantitative (cross-sectional, cohort, before-after, ITS, quasi-experimental); qualitative (interviews, FGDs, case studies, document analyses); mixed-methods; grey literature; outbreak investigation reports with empirical data	Editorials, commentaries, opinion pieces; letters without empirical findings; protocols without results; conference abstracts without full reports; studies published before 2010; non-English or non-French language studies

GHS, Ghana Health Service; IDSR, Integrated Disease Surveillance and Response; DHIMS2, District Health Information Management System 2; GFELTP, Ghana Field Epidemiology and Laboratory Training Programme; FETP, Field Epidemiology Training Program; IHR, International Health Regulations; JEE, Joint External Evaluation; SPAR, State Party Annual Reporting; ITS, Interrupted Time Series; FGDs, Focus Group Discussions; GIS, Geographic Information Systems; SOP, Standard Operating Procedure.

### 2.2 Eligibility criteria

Eligibility criteria are defined using the PICOS framework summarized in Table 1. Full details are provided in the following subsections.

#### 2.2.1 Population

##### 2.2.1.1 Inclusion criteria

Studies will be included if they describe, assess, or evaluate healthcare workforce including field epidemiologists, biostatisticians, disease surveillance officers, public health physicians, laboratory personnel, data managers, and rapid response team members employed by or working with the GHS at national, regional, district, or sub-district levels; or institutional systems including IDSR, DHIMS2, event-based surveillance platforms, outbreak investigation units, public health reference laboratories, the GFELTP including residents and graduates, and epidemic preparedness and response coordination mechanisms; or relevant infrastructure and resources including laboratory diagnostic networks, information technology systems, statistical software, and data management platforms. Exclusion criteria are as listed in Table 1.

#### 2.2.2 Intervention/exposure

Studies will be included if they describe training and capacity-building programs (GFELTP at all levels; biostatistics and statistical modeling training; outbreak investigation simulation exercises; IDSR and data management training); surveillance system interventions (IDSR implementation and strengthening; DHIMS2; electronic and mobile-based reporting; event-based and syndromic surveillance); infrastructure and technology (public health emergency operations centers; laboratory diagnostic capacity; statistical software deployment; Geographic Information Systems; communication technology); and organizational interventions (rapid response team establishment; coordination mechanisms; Standard Operating Procedures; supportive supervision). Both process and outcome evaluations will be considered.

#### 2.2.3 Comparator

This review will include both comparative and non-comparative studies. Where comparators exist, they may include temporal comparisons (pre/post-intervention assessments, longitudinal capacity trends), international standards and benchmarks (WHO IHR core capacity requirements, JEE scoring thresholds, SPAR benchmarks, WHO AFRO regional standards), geographic comparisons (inter-regional, urban versus rural, national versus regional versus district levels), intervention comparisons (trained versus untrained personnel, regions with versus without GFELTP graduates), and control groups where applicable. Studies without formal comparators, including descriptive assessments and situational analyses, will also be included.

#### 2.2.4 Outcomes

The primary and secondary outcomes, with specific indicators and proxies for measurement, are summarized in Table 2.

**Table 2:** Primary and secondary outcomes with measurement indicators and proxies.

Outcome	Category	Indicators and measurement proxies	Measurement tools and data sources
1. Overall field epidemiology preparedness level	Primary	JEE core capacity scores (scale 1–5 per 19 technical areas); SPAR indicator scores (scale 1–100); IHR self-assessment scores; composite preparedness indices	WHO JEE reports; State Party Annual Reports; IHR monitoring framework data; national assessments
2. Surveillance system performance and outbreak response capacity	Primary	IDSR reporting completeness (% of expected reports received); IDSR timeliness (% of reports submitted within deadline); outbreak detection time (days from onset to detection); proportion of outbreaks investigated within 48 h; proportion with laboratory confirmation; DHIMS2 data quality scores	GHS annual surveillance reports; DHIMS2 data; outbreak investigation reports; WHO AFRO surveillance bulletins
3. Field epidemiology workforce capacity	Secondary	Number and density of trained field epidemiologists per 100,000 population; GFELTP completion rates; post-training competency assessment scores; trainee deployment rates to intended positions; graduate retention in field epidemiology roles at 1, 3, and 5 years; geographic distribution of graduates	GFELTP program reports; GHS workforce registries; AFENET/TEPHINET databases
4. Statistical modeling and biostatistical capacity	Secondary	Number of qualified biostatisticians per 100,000 population; availability and utilization rates of statistical software (Epi Info, R, STATA, SPSS); documented use of mathematical or compartmental models for outbreak projections; number of published epidemiological analyses using quantitative modeling methods; integration of statistical evidence into policy documents; number of DHIMS2-generated analytical reports used for programmatic decision-making	GHS reports; academic publications; institutional assessments; program evaluations
5. Infrastructure, governance, and data systems	Secondary	Number and functionality of public health laboratories with diagnostic capacity for priority diseases; DHIMS2 implementation status and utilization rates; electronic surveillance platform adoption rates; mobile reporting system coverage; existence of updated Standard Operating Procedures for outbreak investigation; policy documents integrating statistical evidence; multi-sectoral coordination mechanisms	GHS infrastructure assessments; laboratory accreditation records; policy document reviews; institutional audits

##### 2.2.4.1 Notes on outcome 4 (statistical modeling and biostatistical capacity)

In the absence of direct measures of statistical modeling practice, the following proxies will be used: availability and utilization of statistical software (as a proxy for analytical infrastructure); publications of epidemiological analyses from GHS data (as a proxy for analytical output); biostatistician-to-population ratios (as a proxy for workforce capacity); and documented incorporation of quantitative evidence into outbreak response and policy documents (as a proxy for integration of analytical capacity into decision-making processes). These proxies will be interpreted with appropriate caution regarding the gap between capacity availability and actual practice, which will be addressed in the narrative synthesis.

#### 2.2.5 Study designs

##### 2.2.5.1 Inclusion criteria

The following study designs will be included: quantitative studies including cross-sectional surveys, cohort studies (prospective or retrospective), before-after evaluations, Interrupted Time Series analyses, quasi-experimental studies, randomized controlled trials if available, and surveillance system evaluations using standardized tools such as the Joint External Evaluation, SPAR, and IHR monitoring frameworks; qualitative studies including in-depth interviews, Focus Group Discussions, case studies, ethnographic studies, and document analyses examining perceptions, experiences, and contextual factors related to field epidemiology preparedness; mixed-methods studies combining quantitative and qualitative approaches; implementation research evaluating processes, barriers, facilitators, and outcomes of preparedness interventions; grey literature including government reports, technical assessments, policy documents, program evaluations, situation analyses, JEE and after-action review reports, training program reports, WHO assessments, and institutional reports containing empirical data; and outbreak investigation reports containing empirical data on field epidemiology capacity and response.

Outbreak investigation reports are specifically included because they represent primary empirical documentation of field epidemiology capacity in action, capturing real-world data on detection timeliness, investigative methods, workforce deployment, laboratory confirmation, and response effectiveness that may not be available through other study designs in this resource-limited setting (7, 25). This justification is consistent with WHO guidance on evidence synthesis for public health systems research.

##### 2.2.5.2 Exclusion criteria

The following will be excluded: editorials, commentaries, and opinion pieces without original data; letters to editors without empirical findings; systematic reviews and meta-analyses (though their reference lists will be searched); protocols without results; conference abstracts without full reports; studies published before January 1, 2010; and studies in languages other than English or French.

Different study type integration: Quantitative studies will be narratively synthesized using tabulation of key metrics and, where appropriate, pooled in meta-analyses. Qualitative studies will be thematically synthesized to identify patterns in implementation barriers, enablers, and contextual factors. Mixed-methods studies will contribute findings from both quantitative and qualitative components to the respective syntheses. Grey literature and outbreak reports will be analyzed alongside peer-reviewed evidence to assess consistency and completeness of the evidence base. The quality of each study type will be assessed using appropriate tools, and the contribution of each study type to the overall narrative will be clearly differentiated in the synthesis.

#### 2.2.6 Context and setting

This review focuses on the Ghana Health Service across all 16 administrative regions including Greater Accra, Ashanti, Central, Western, Eastern, Volta, Northern, Upper East, Upper West, Brong Ahafo, Savannah, North East, Bono East, Oti, Ahafo, and Western North. Studies conducted at national, regional, district, or sub-district levels will be eligible. Studies published from January 1, 2010 to present are included to capture key developments including WHO IHR 2005 implementation, GFELTP maturation (2007 to present), IDSR strengthening, Ebola epidemic preparedness (2014 to 2016), and COVID-19 pandemic response (2020 to present).

### 2.3 Information sources

#### 2.3.1 Electronic bibliographic databases

The following databases will be searched from January 1, 2010 to present: PubMed/MEDLINE via the PubMed interface; African Index Medicus via WHO AFRO; and African Journals Online (AJOL). Google Scholar will be searched (first 200 results) to capture grey literature. Subscription limitations have restricted access to Scopus, Web of Science, EMBASE, and Global Health Database; this constraint is acknowledged as a potential limitation to comprehensiveness and is documented transparently. Each database will be searched individually using database-specific syntax and, where available, controlled vocabulary (MeSH terms for PubMed; subject headings for AIM and AJOL).

#### 2.3.2 Grey literature sources

Institutional websites and repositories searched will include the GHS website and document repository; Ministry of Health Ghana publications; GFELTP program documents; University of Ghana School of Public Health repository; and Noguchi Memorial Institute for Medical Research publications. International organizations searched will include the WHO AFRO Regional Office; WHO Ghana country office; Joint External Evaluation reports for Ghana; State Party Annual Reports to WHO; West African Health Organisation; CDC Ghana office reports; USAID Ghana health sector documents; African Field Epidemiology Network (AFENET); and TEPHINET. Thesis and dissertation repositories will include ProQuest Dissertations and Theses, the University of Ghana digital repository, the University of Cape Coast repository, and the Kwame Nkrumah University of Science and Technology repository.

#### 2.3.3 Other sources

Reference lists of all included studies will be hand-searched (backward citation searching), and studies citing included studies will be identified through forward citation searching using Google scholar. Experts including GHS disease surveillance department staff, GFELTP program coordinators, and WHO Ghana country office personnel will be contacted for unpublished data or grey literature not captured in database searches.

### 2.4 Search strategy

The core search strategy was developed around three main concept areas: (1) Ghana and geographic terms; (2) Ghana Health Service, public health systems; and (3) preparedness, field epidemiology, surveillance, and biostatistics. Search terms were developed by reviewing key papers to identify relevant terminology, using MeSH terms and database-specific controlled vocabularies, and applying Boolean operators (OR to combine synonyms within concepts, AND to combine concepts, asterisk truncation to capture word variations, and quotation marks for phrase searching). The strategy was pilot-tested to ensure known relevant studies were captured.

Full search strategies for all databases are provided in the supplementary materials. The consolidated PubMed search strategy is provided below as an example.

#### 2.4.1 PubMed consolidated search string

(Ghana[tiab] OR Ghanaian[tiab] OR “West Africa”[tiab] OR “West African”[tiab]) AND (“health service”[tiab] OR “health system”[tiab] OR “public health”[tiab] OR “Ghana Health Service”[tiab] OR “GHS”[tiab] OR “Ministry of Health”[tiab] OR “Disease Surveillance Department”[tiab]) AND (preparedness[tiab] OR readiness[tiab] OR capacity[tiab] OR capability[tiab] OR competency[tiab] OR strengthening[tiab]) AND (“field epidemiology”[tiab] OR epidemiology[tiab] OR FELTP[tiab] OR GFELTP[tiab] OR “field epidemiology training”[tiab] OR “Field Epidemiology and Laboratory Training”[tiab] OR FETP[tiab] OR surveillance[tiab] OR “disease surveillance”[tiab] OR “public health surveillance”[tiab] OR IDSR[tiab] OR “Integrated Disease Surveillance and Response”[tiab] OR DHIMS2[tiab] OR outbreak[tiab] OR “outbreak investigation”[tiab] OR “outbreak response”[tiab] OR biostatistics[tiab] OR “statistical modeling”[tiab] OR “statistical model”[tiab] OR “mathematical model”[tiab] OR forecasting[tiab] OR “Epi Info”[tiab] OR IHR[tiab] OR “International Health Regulations”[tiab] OR “Joint External Evaluation”[tiab] OR JEE[tiab] OR “global health security”[tiab] OR SPAR[tiab] OR “core capacity”[tiab] OR “laboratory capacity”[tiab] OR infrastructure[tiab] OR “Epidemiology”[Mesh] OR “Population Surveillance”[Mesh] OR “Disease Outbreaks”[Mesh]) AND (“2010/01/01”[Date - Publication]: “3000”[Date - Publication]) AND (English[lang] OR French[lang])

The search will be updated approximately 2–4 weeks before final manuscript submission to capture newly published studies.

### 2.5 Study selection process

#### 2.5.1 Citation management

All search results will be exported to EndNote 20 reference management software. Duplicate citations will be identified and removed using EndNote’s duplicate detection function, followed by manual verification. The deduplication process will be documented, including total citations imported, duplicates removed, and unique citations proceeding to screening.

#### 2.5.2 Screening process

Covidence systematic review software will be used to manage the screening process (38).

*Stage 1: Title and Abstract Screening:* Two reviewers will independently screen titles and abstracts against eligibility criteria, classifying each citation as include, exclude, or uncertain. Inter-rater reliability will be calculated using Cohen’s kappa statistic with a target of 0.70 or above (39). Disagreements will be resolved through discussion with consultation with a third reviewer if consensus is not reached.

*Stage 2: Full-Text Screening:* Full-text articles of all citations marked include or uncertain will be retrieved. Two reviewers will independently assess full texts against eligibility criteria using a standardized Covidence form. Reasons for exclusion will be documented using predefined categories (wrong population, wrong intervention, wrong outcome, wrong study design, wrong geographic setting, wrong time period, full text not available, duplicate, and other). Inter-rater agreement will be calculated. Articles where full texts cannot be obtained after up to three email attempts over 4 weeks will be listed in a Studies Awaiting Classification section.

A PRISMA 2020 flow diagram will document citations identified from each database, duplicates removed, citations screened at title and abstract stage, citations excluded, full-text articles assessed for eligibility, full-text articles excluded with reasons, and studies included in quantitative and qualitative synthesis.

### 2.6 Data collection process

A standardized data extraction form will be developed in Microsoft Excel covering study identification, study characteristics, population, intervention and exposure details, comparator details, outcome data, risk of bias assessment items, and notes. The form will be pilot-tested on five diverse studies by both reviewers independently and refined for clarity, completeness, and inter-rater agreement. Two reviewers will independently extract data from all included studies, with disagreements resolved through discussion or third reviewer consultation. Corresponding authors will be contacted for missing or unclear data.

### 2.7 Risk of bias and quality assessment

Risk of bias assessment tools will be selected according to study design, ensuring methodological appropriateness. A summary of tools and key assessment domains is provided in Table 3 (56–59).

**Table 3:** Risk of bias and quality assessment tools by study design.

Study type	Assessment tool	Key domains assessed	Rating scale
Cross-sectional studies (analytical and descriptive)	JBI Critical Appraisal Checklist for Analytical Cross-Sectional Studies (8 items)	Inclusion criteria; description of subjects and setting; valid and reliable measurement of exposure and outcome; appropriate statistical analysis; identification and management of confounders	Yes/no/unclear per item; overall low/moderate/high risk
Qualitative studies	CASP Qualitative Checklist (10 items)	Clarity of aims; appropriate methodology; appropriate research design; appropriate recruitment strategy; data collection methods; reflexivity; ethical considerations; data analysis rigor; clarity of findings; value of research	Yes/no/cannot tell per item
Mixed-methods studies	Mixed Methods Appraisal Tool (MMAT) Version 2018 (25 items)	Quantitative component: sampling, measurement, non-response; qualitative component: appropriateness, analytical process; mixed-methods integration justification	Yes/no/cannot tell per item
Cohort and before-after evaluation studies	Newcastle–Ottawa Scale adapted for non-randomized studies	Selection of study groups; comparability of groups on key factors; assessment and ascertainment of outcome; adequacy of follow-up	0–9 stars; low risk (7, 8, 13), moderate (4–6), high risk (0–3)
Grey literature (government reports, institutional assessments, program evaluations)	AACODS Checklist (Authority, Accuracy, Coverage, Objectivity, Date, Significance)	Authorship authority; accuracy and evidence base; coverage and scope; objectivity and potential bias; currency; significance to research question	High/medium/low for each criterion
Outbreak investigation reports	Adapted JBI Checklist for Prevalence Studies (modified for field epidemiology reports)	Case definition clarity; representativeness of sample; validity of data collection methods; completeness of reporting; appropriateness of analysis; conclusions consistent with data	Yes/no/unclear per item

Two reviewers will independently assess risk of bias for all included studies. Disagreements will be resolved through discussion with consultation with a third reviewer. Overall quality will be classified as high quality (low risk of bias across most domains), moderate quality (some concerns not seriously compromising validity), or low quality (high risk of bias in multiple domains). Studies will not be excluded based on quality alone; rather, sensitivity analyses will explore the impact of study quality on conclusions. Risk of bias results will be presented in a summary table showing assessments across all domains and a risk of bias graph showing the distribution of low, moderate, and high risk for each domain. The plan for integrating risk-of-bias results into the synthesis is as follows: studies rated as high quality will form the primary evidence base for conclusions; studies of moderate quality will be used where high-quality evidence is absent, with appropriate caveats; studies of low quality will be included in a sensitivity analysis only. Discordance between quality tiers will be highlighted in the narrative synthesis as a source of uncertainty.

### 2.8 Data synthesis

#### 2.8.1 Narrative synthesis

Narrative synthesis will be conducted for all included studies following the framework by Popay et al. (40) and the Cochrane Handbook (41). The synthesis will develop a preliminary logic model of field epidemiology preparedness, organize studies by thematic domains corresponding to the five outcome areas in Table 2, explore patterns across studies examining consistency, contradictions, moderating factors, and contextual influences, and assess the robustness of findings considering quality of included studies. Findings will be mapped against the WHO IHR core capacity framework (1) to identify specific gaps classified by severity, domain, geographic location, and health system level.

For studies that yield exclusively descriptive qualitative outputs, the following mitigation strategy will be applied: qualitative findings will be synthesized thematically using framework analysis aligned with the WHO IHR core capacity domains, enabling structured comparison across qualitative studies even where quantitative data are absent. Convergent synthesis will be used to integrate qualitative and quantitative evidence on the same outcome domains, explicitly noting where qualitative findings corroborate, extend, or contradict quantitative data. Where only qualitative evidence is available for a given domain, findings will be reported with appropriate hedging and designated as low to very low certainty evidence under the adapted GRADE framework.

#### 2.8.2 Meta-analysis

Narrative synthesis is anticipated to be the primary analytic approach given the expected diversity of study designs, populations, and outcome measurement methods in this under-researched field. Meta-analysis will be considered if three or more comparable studies report the same outcome using consistent measurement methods and demonstrate acceptable clinical and methodological homogeneity following data extraction. Specific criteria for meta-analytic feasibility will be evaluated at the data synthesis stage. Where quantitative pooling is conducted, random-effects models using the DerSimonian–Laird method will be used as the primary approach, with appropriate effect measures selected by data type (risk ratios or odds ratios for dichotomous outcomes, mean difference or standardized mean difference for continuous outcomes, pooled proportions with 95% confidence intervals for prevalence data). Statistical heterogeneity will be assessed using the *I*^2^ statistic and Chi-squared test. Where substantial heterogeneity (*I*^2^ greater than 50%) is found, subgroup analyses and meta-regression will be explored to identify sources of variation.

#### 2.8.3 Subgroup and sensitivity analyses

Planned subgroup analyses will examine geographic region, health system level, time period, study design, and urban versus rural settings, subject to availability of three or more studies per subgroup. Sensitivity analyses will include exclusion of studies with high risk of bias, exclusion of grey literature, and comparison of fixed-effect versus random-effects models.

#### 2.8.4 Assessment of reporting bias

Funnel plots and Egger’s regression test will be used to assess publication bias if 10 or more studies are included in a meta-analysis (42). Other reporting biases will be assessed by comparing outcomes listed in methods versus results sections and by comparing grey literature findings with peer-reviewed publications. Mitigation strategies include comprehensive grey literature search, contact with GHS for unpublished reports, and author contact for missing data.

### 2.9 Confidence in cumulative evidence

Evidence certainty will be assessed using an adapted GRADE framework appropriate for public health systems research (43). For each main outcome, evidence will be rated and downgraded based on risk of bias, inconsistency, indirectness, imprecision, and publication bias. Upgrading will be considered for large magnitude of effect, dose–response gradient, and plausible confounding. Evidence will be classified as high, moderate, low, or very low certainty. Summary of Findings tables will present evidence for each main outcome with overall certainty ratings.

## 3 Discussion

### 3.1 Expected findings

This systematic review is expected to provide the first comprehensive synthesis of evidence on Ghana Health Service preparedness for field epidemiology and applied biostatistics. Evidence will be summarized across five thematic domains corresponding to the outcomes in Table 2: (1) overall preparedness as measured by JEE and SPAR scores; (2) surveillance system performance and outbreak response capacity; (3) field epidemiology workforce capacity; (4) statistical modeling and biostatistical capacity; and (5) infrastructure, governance, and data systems.

Within these domains, we anticipate finding evidence of progressive strengthening of field epidemiology capacity over the 2010 to 2025 period, particularly following major epidemic events such as the 2014 to 2016 Ebola outbreak and the COVID-19 pandemic. The GFELTP is expected to have produced a growing cadre of trained field epidemiologists, though geographic distribution, retention, and deployment of graduates to areas of greatest need remain uncertain (24–26). Surveillance system performance is expected to show improvement over time with IDSR and DHIMS2 strengthening, but persistent challenges in reporting completeness and timeliness at lower health system levels and in resource-constrained regions are anticipated (21, 31). Statistical modeling and biostatistical capacity is expected to be concentrated at national and regional levels, with limited capacity at district levels (8, 30). Infrastructure and technology gaps, particularly in laboratory capacity, transportation for field investigations, and digital connectivity for real-time reporting, are anticipated as persistent challenges.

Where studies yield exclusively descriptive qualitative outputs without quantitative data, findings will be incorporated through thematic framework analysis aligned with WHO IHR core capacity domains. This approach allows structured comparison across qualitative studies and explicit integration with quantitative evidence where both exist. The risk of quantitative-qualitative discordance will be mitigated by separate sub-synthesis and convergence mapping, with clear reporting of the certainty of evidence and areas where conclusions rest predominantly on qualitative rather than quantitative evidence. This approach is consistent with established guidance for mixed-evidence synthesis in health systems research (40, 41).

Geographic variation is expected to be a significant finding, with Northern and rural regions likely demonstrating lower preparedness capacity relative to Southern and urban areas due to infrastructure and workforce distribution disparities (8, 21). Substantial heterogeneity across studies in study design, outcome measurement, and geographic focus is anticipated and will be addressed through transparent reporting of heterogeneity statistics and subgroup analyses.

### 3.2 Implications for policy and practice

Findings from this review will have important implications for policy and practice in Ghana and similar resource-limited settings. The comprehensive gap analysis mapped against WHO IHR core capacities will provide specific, actionable recommendations for strengthening epidemic preparedness. For the GHS and Ministry of Health, the review will inform strategic planning, guide resource allocation, identify priority areas for international partnerships, and support advocacy for sustained funding for field epidemiology and surveillance systems. For the GFELTP and other training programs, findings will guide curriculum development, workforce planning, deployment and retention strategies, and continuing professional development priorities. For development partners and international organizations, findings will guide targeting of technical and financial assistance, inform regional approaches to capacity strengthening, and support harmonization with national priorities.

### 3.3 Implications for research

This systematic review will identify important gaps in the evidence base warranting further research, including limited rigorous evaluations of training program long-term effectiveness, insufficient evidence on cost-effectiveness of capacity-building interventions, inadequate documentation of field epidemiology integration with broader health system strengthening, and limited evidence on equity dimensions of preparedness across urban and rural areas. Future research priorities include longitudinal studies tracking GFELTP graduates; implementation research on barriers to deployment; cost-effectiveness analyses; studies examining innovative approaches to workforce and infrastructure gaps; and evaluations of digital health technologies for surveillance in limited-connectivity contexts.

### 3.4 Strengths and limitations

#### 3.4.1 Strengths

This systematic review will be the first comprehensive synthesis of evidence on field epidemiology preparedness specific to Ghana. It employs rigorous methodology following PRISMA 2020 guidelines with PROSPERO registration, a comprehensive multi-database search strategy including grey literature and expert consultation, inclusion of diverse study designs to provide a holistic understanding of preparedness, multiple quality assessment tools appropriate for different study designs, independent dual review at all stages, and mapping of findings to the WHO IHR core capacity framework for internationally standardized gap assessment.

#### 3.4.2 Limitations

This review will have several limitations that should be acknowledged. The quality and quantity of available evidence may be limited given the under-researched nature of this field in the Ghanaian context. Publication bias may exist if negative findings or implementation challenges are less likely to be published. Grey literature quality may be variable, which will be addressed through systematic AACODS quality assessment. Language restrictions to English and French may exclude relevant studies, though these are the predominant publication languages in West Africa. Subscription limitations have restricted access to Scopus, Web of Science, and EMBASE, which may reduce comprehensiveness.

Substantial heterogeneity across included studies is anticipated in terms of study designs, populations, geographic scope, time periods, and outcome measurement methods. This methodological heterogeneity may limit the feasibility of quantitative pooling and complicate cross-study comparisons, and will be addressed through transparent subgroup analyses and sensitivity analyses as described in section 2.8. Proxy bias is an acknowledged limitation: several outcomes, particularly statistical modeling capacity and overall preparedness, will necessarily be measured using proxy indicators (such as software availability, JEE scores, and publication counts) rather than direct assessments of analytical practice or capacity performance. These proxies may not fully capture the true construct of interest, and their limitations will be explicitly discussed in the interpretation of findings. The cross-sectional nature of many expected studies limits causal inference. The focus on Ghana limits direct generalizability, though the methodology is replicable in similar resource-limited settings.

### 3.5 Significance and innovation

Despite these limitations, this systematic review represents an important contribution to the field of global health security and epidemic preparedness in resource-limited settings. The comprehensive synthesis of fragmented evidence will provide the most complete picture to date of Ghana’s field epidemiology capacity. The use of the WHO IHR framework for gap analysis provides an internationally recognized standard for assessment. The inclusion of statistical modeling and biostatistical capacity alongside traditional field epidemiology domains reflects the increasing importance of quantitative methods for epidemic preparedness in LMICs (8). The replicable methodology will allow other countries to conduct similar assessments, contributing to regional and global health security evidence.

## 4 Ethics and dissemination

### 4.1 Ethical considerations

This systematic review does not require ethical approval as it involves secondary analysis of published literature and does not involve direct contact with human participants. All included studies will have undergone their own ethical review processes as appropriate. Proper attribution of all sources will be ensured. Any potential conflicts of interest will be declared transparently.

### 4.2 Dissemination plan

Results will be disseminated through peer-reviewed publication in Frontiers in Public Health as the primary target journal, with open access publication to ensure availability to researchers, policymakers, and practitioners in Ghana and across Africa. Alternative target journals include BMC Public Health, PLOS ONE, Global Health Action, and African Health Sciences. The review will also form a chapter of an MPhil thesis at the University of Cape Coast and will be deposited in the university repository.

Conference presentations will include the Ghana Health Service Annual Research Conference, AFENET Scientific Conference, TEPHINET Global Scientific Conference, and the West African Health Organisation conferences. A policy brief will be prepared and distributed to the GHS Director-General, Disease Surveillance Department, Ministry of Health, GFELTP Program leadership, WHO Ghana Country Office, and development partners including CDC, USAID, and AFENET. Consistent with open science principles, the review protocol is publicly available on PROSPERO, and data extraction files, search strategies, and analysis code will be deposited in an open repository upon completion.
