# Supplementary material for: Preparedness of the Ghana Health Service for field epidemiology and applied biostatistics: a systematic review protocol of infectious disease surveillance, outbreak investigation methodologies, and statistical modeling capacities in resource-limited settings
Source: Front Public Health. 2026 Jun 12;14:1803063. doi: 10.3389/fpubh.2026.1803063 (PMC13303809; doi:10.3389/fpubh.2026.1803063)
Supplement: Supplementary file 1 [file Data_Sheet_1.PDF]

NIHR | National Institute for Health and Care Research

PROSPERO
International prospective register of systematic reviews

Preparedness of the Ghana Health Service for Field Epidemiology and Applied Biostatistics: A Systematic Review of Infectious Disease Surveillance, Outbreak Investigation Methodologies, and Statistical Modeling Capacities in Resource-Limited Settings

Victor Luckyboy Dzramado, William Wilberforce Amoah, Samuel Antwi, Joana Edem Koto, Doris Hagan

Citation

Victor Luckyboy Dzramado, William Wilberforce Amoah, Samuel Antwi, Joana Edem Koto, Doris Hagan. Preparedness of the Ghana Health Service for Field Epidemiology and Applied Biostatistics: A Systematic Review of Infectious Disease Surveillance, Outbreak Investigation Methodologies, and Statistical Modeling Capacities in Resource-Limited Settings. PROSPERO 2026 CRD420261299788. Available from https://www.crd.york.ac.uk/PROSPERO/view/CRD420261299788.

REVIEW TITLE AND BASIC DETAILS

Review title

Preparedness of the Ghana Health Service for Field Epidemiology and Applied Biostatistics: A Systematic Review of Infectious Disease Surveillance, Outbreak Investigation Methodologies, and Statistical Modeling Capacities in Resource-Limited Settings

Condition or domain being studied

Signs/symptoms-physical Surveillance; Disease Outbreak; Public health practice; Epidemic ; Healthcare Professional ; Surveillance

This systematic review examines the preparedness of Ghana Health Service for field epidemiology and applied biostatistics within three interconnected domains: infectious disease surveillance systems, outbreak investigation methodologies, and statistical modeling capacity for public health decision-making in resource-limited settings.

The review evaluates preparedness across multiple dimensions including trained workforce

availability and competency (particularly Field Epidemiology and Laboratory Training Program graduates), surveillance system functionality (Integrated Disease Surveillance and Response implementation), laboratory diagnostic capacity, outbreak detection and response mechanisms, data management infrastructure (DHIMS2 and electronic systems), and statistical expertise for evidence-based policy-making.

Assessment will be conducted against WHO International Health Regulations (2005) core capacities, Joint External Evaluation frameworks, and regional standards. The domain encompasses both technical capacity (epidemiological and biostatistical skills, investigation protocols, modeling tools) and operational systems (infrastructure, coordination mechanisms, resource availability) essential for detecting, investigating, and responding to infectious disease threats in Ghana and similar West African contexts.

Rationale for the review

Infectious disease outbreaks continue to pose significant threats to global health security, with resource-limited settings in West Africa being particularly vulnerable. Recent epidemics including Ebola, COVID-19, and endemic diseases like cholera and meningitis have highlighted critical gaps in preparedness and response capacity.

The Ghana Health Service plays a central role in disease surveillance and outbreak response in Ghana and the West African sub-region. Despite investments in Field Epidemiology and Laboratory Training Programs (FELTP) and surveillance system strengthening, there has been no comprehensive systematic synthesis of Ghana's preparedness for field epidemiology and applied biostatistics.

Current evidence remains fragmented across grey literature, programme evaluations, and isolated studies, making it difficult for policymakers to identify specific capacity gaps and prioritize interventions. While individual assessments exist, no systematic review has comprehensively evaluated the integration of surveillance systems, outbreak investigation capacity, and statistical modeling capabilities within the Ghana Health Service context.

This review will fill this critical knowledge gap by systematically synthesizing available evidence on Ghana's field epidemiology preparedness across three interconnected domains: surveillance, outbreak investigation, and biostatistical capacity. It will provide the first comprehensive assessment of strengths, weaknesses, and gaps aligned with WHO International Health Regulations core capacities.

Findings will inform evidence-based policy decisions, guide resource allocation, identify training priorities, and provide a replicable framework for assessing field epidemiology preparedness in similar resource-limited settings across Africa. This timely synthesis will contribute to strengthening epidemic preparedness and advancing global health security objectives.

Review objectives

This systematic review aims to comprehensively assess the preparedness of the Ghana Health Service for field epidemiology and applied biostatistics. Specific review questions are:

Primary Objectives:

1. What is the current state of preparedness of the Ghana Health Service for infectious disease surveillance and outbreak investigation?

2. What statistical modeling and biostatistical capacities exist within the Ghana Health Service for evidence-based public health decision-making?

3. What gaps exist in Ghana Health Service preparedness compared to WHO International Health Regulations (IHR) core capacities and international standards for field epidemiology?

Secondary Objectives:

1. What training interventions and capacity-building programs have been implemented to strengthen field epidemiology and biostatistics workforce in Ghana, and what are their outcomes?

2. What infrastructure, resources, and systems (surveillance platforms, laboratory networks, data management systems) are available to support infectious disease surveillance, outbreak investigation, and statistical analysis?

3. How does Ghana Health Service preparedness compare across different geographical regions and health system levels (national, regional, district)?

4. What policy and programmatic recommendations can be made to address identified gaps and strengthen field epidemiology capacity in resource-limited settings?

Keywords

Ghana Health Service; field epidemiology; applied biostatistics; Infectious disease surveillance; Outbreak investigation; Statistical Modeling; public health preparedness; Resource-limited settings; West Africa; epidemic preparedness; disease surveillance systems; public health capacity; health workforce training; Integrated Disease Surveillance and Response; Field Epidemiology and Laboratory Training Program; outbreak response capacity; epidemiological capacity assessment; Health systems strengthening; laboratory capacity; Public health decision-making

Country

Ghana

ELIGIBILITY CRITERIA

Population

Included
Studies will be included if they describe, assess, or evaluate:

Healthcare Workforce: Field epidemiologists, biostatisticians, disease surveillance officers, public health physicians, laboratory personnel, data managers, rapid response team members, and other public health professionals employed by or working with the Ghana Health Service at national, regional, district, or sub-district levels.

Institutional Systems: Ghana Health Service surveillance systems including Integrated Disease

Surveillance and Response (IDSR), District Health Information Management System (DHIMS2), event-based surveillance platforms, outbreak investigation units, public health reference laboratories, the Ghana Field Epidemiology and Laboratory Training Program (GFELTP) including residents and graduates, and epidemic preparedness and response coordination mechanisms.

Infrastructure and Resources: Laboratory diagnostic networks, information technology systems supporting disease surveillance, statistical software and analytical tools, communication systems for disease reporting and outbreak alerts, transportation resources for field investigations, and data management platforms.

Studies focusing on infectious disease surveillance capacity, outbreak investigation preparedness, statistical modeling capabilities, training program outcomes, workforce competency assessments, system performance evaluations, or infrastructure assessments within the Ghana Health Service context will be eligible.

Excluded
Studies will be excluded if they:

Focus exclusively on clinical management of diseases without surveillance or epidemiological components

Describe health services outside Ghana without comparative data

Focus solely on primary healthcare delivery unrelated to disease surveillance or outbreak response

Describe only community health workers or volunteers without Ghana Health Service linkage

Focus exclusively on maternal-child health, non-communicable diseases, or mental health programs without infectious disease surveillance components

Are conducted in private healthcare facilities without Ghana Health Service involvement

Intervention(s) or exposure(s)

Included
Training; Signs/symptoms-physical Surveillance; Laboratory procedure; Laboratory Equipment; Following Protocol; Routine Health Information System; Skills training; Emergency Treatment; Emergency Department Patient Visit; Emergency Care

Interventions and exposures of interest include:

Training and Capacity Building: Ghana Field Epidemiology and Laboratory Training Program (GFELTP) including two-year advanced program, three-month frontline program, and short-term training courses; biostatistics and statistical modeling training; outbreak investigation simulation exercises; continuing professional development programs.

Surveillance System Interventions: Implementation and strengthening of Integrated Disease Surveillance and Response (IDSR); electronic surveillance platforms (DHIMS2, mobile-based reporting); event-based surveillance mechanisms; laboratory-based surveillance networks;

syndromic surveillance systems.

Infrastructure and Technology: Public health emergency operations centers; laboratory diagnostic capacity enhancement; statistical software deployment (Epi Info, R, STATA, SPSS); geographic information systems for disease mapping; communication technology for real-time reporting.

Organizational Interventions: Rapid response team establishment; epidemic preparedness committees; coordination mechanisms between health system levels; standard operating procedures for outbreak investigation; data quality improvement initiatives.

Studies describing existing capacity levels, implementation processes, effectiveness evaluations, or assessments of these interventions will be included. Both process and outcome evaluations will be considered.

Excluded
Interventions focusing exclusively on:

Clinical treatment protocols without surveillance components

Vaccination campaigns without surveillance system strengthening

General health system reforms unrelated to disease surveillance

Medical equipment procurement unrelated to laboratory diagnostics or epidemiological investigation

Administrative reforms without field epidemiology focus

Comparator(s) or control(s)

Included
PICO tags selected: Surveillance; Active Surveillance; Screening Surveillance; Surveillance system

Where comparative studies exist, the following comparators will be included:

Temporal Comparisons: Pre-intervention baseline measurements compared with post-intervention assessments; longitudinal trends showing changes in capacity over time (e.g., surveillance performance 2010-2015 vs. 2016-2025).

International Standards and Benchmarks: WHO International Health Regulations (IHR) core capacity requirements; Joint External Evaluation (JEE) scoring thresholds; State Party Annual Reporting (SPAR) benchmarks; WHO AFRO regional standards for disease surveillance and outbreak response; global best practices for field epidemiology programs.

Geographic Comparisons: Comparisons between Ghana and other West African countries with similar resource contexts; comparisons across Ghana's 16 regions; urban versus rural preparedness comparisons; national versus regional versus district level capacity comparisons.

Intervention Comparisons: Trained versus untrained personnel; facilities with enhanced surveillance systems versus standard systems; regions with FELTP graduates versus those without; pre-FELTP versus post-FELTP implementation periods.

Control Groups: For intervention studies, usual practice or no-intervention control groups; waitlist controls; alternative intervention comparisons.

Studies without formal comparators (descriptive assessments, situational analyses) will also be included to comprehensively capture available evidence.

Excluded
Studies will be excluded if comparators are:

From high-income countries with substantially different health system contexts that limit applicability to Ghana's resource-limited setting

Based on outdated international standards that have been superseded (e.g., pre-IHR 2005 frameworks)

Using non-validated or inappropriate assessment tools

Comparing Ghana with countries outside Africa without adjustment for contextual differences

Using comparators that are inadequately described or measured, preventing meaningful interpretation of comparative findings

Study design

Both randomized and nonrandomized study types will be included.

Included
The following study designs will be included:

Quantitative Studies: Cross-sectional surveys, cohort studies (prospective or retrospective), before-after evaluations, interrupted time series, quasi-experimental studies, randomized controlled trials (if available), surveillance system evaluations, capacity assessments using standardized tools (Joint External Evaluation, State Party Annual Reporting, IHR monitoring frameworks).

Qualitative Studies: In-depth interviews, focus group discussions, case studies, ethnographic studies, document analyses examining perceptions, experiences, and contextual factors related to field epidemiology preparedness.

Mixed-Methods Studies: Studies combining quantitative and qualitative approaches to evaluate preparedness, training programs, or system implementation.

Implementation Research: Studies evaluating implementation processes, barriers, facilitators, and outcomes of preparedness interventions.

Grey Literature: Government reports, technical assessments, policy documents, program evaluations, situation analyses, Joint External Evaluations, after-action reviews of outbreak responses, training program reports, WHO assessments, and institutional reports containing empirical data.

Outbreak Investigation Reports: Published outbreak investigations demonstrating field epidemiology capacity and response.

No restrictions will be placed on study design to capture the full breadth of available evidence on Ghana Health Service preparedness in this under-researched area.

Excluded
Types of Study Excluded (68 words):

The following will be excluded:

Editorials, commentaries, and opinion pieces without original data

Letters to editors without empirical findings

Systematic reviews and meta-analyses (although reference lists will be searched)

Protocols without results

Conference abstracts without full reports (unless authors provide additional data)

Studies published before 2010

Studies in languages other than English or French

Context

This review focuses on the Ghana Health Service, the principal implementer of public health services in Ghana, a lower-middle-income country in West Africa with a population of approximately 33 million people.

Geographic Scope: All 16 administrative regions of Ghana, including urban and rural settings. Studies may focus on national-level systems, regional health directorates, district health management teams, or sub-district facilities.

Health System Context: Ghana operates a decentralized health system with the Ghana Health Service responsible for disease surveillance, outbreak investigation, and epidemic response. The system includes national public health reference laboratories, regional disease surveillance units, and district-level rapid response teams.

Time Period: Studies published from January 2010 to present will be included. This period captures implementation of revised International Health Regulations (2005), establishment of Ghana FELTP (2007-present), Ebola epidemic preparedness (2014-2016), and COVID-19 pandemic response (2020-present).

Disease Context: Focus on infectious diseases of public health importance including epidemic-prone diseases (cholera, cerebrospinal meningitis, yellow fever, viral hemorrhagic fevers), vaccine-preventable diseases, zoonotic diseases, emerging infections, and diseases under Integrated Disease Surveillance and Response (IDSR).

Resource Setting: Recognition of resource-limited setting constraints including limited laboratory infrastructure, workforce shortages, inadequate transportation, technology gaps, and funding limitations.

Studies from other West African countries will be included only if they provide direct comparative data with Ghana or regional context relevant to understanding Ghana's preparedness.

TIMELINE OF THE REVIEW

Date of first submission to PROSPERO
03 February 2026

Review timeline
Start date: 1 September 2025. End date: 31 March 2026.

Date of registration in PROSPERO
03 February 2026

AVAILABILITY OF FULL PROTOCOL

Availability of full protocol
A full protocol has been written and uploaded to PROSPERO. The protocol will be made available after the review is completed.

SEARCHING AND SCREENING

Search for unpublished studies
Only published studies will be sought.

Main bibliographic databases that will be searched
The main databases to be searched are *MEDLINE*, *PubMed* and *Scopus*.

Search language restrictions
The review will only include studies published in English.

Search date restrictions
Databases will be searched for articles published from 1 January 2010 and before by 31 December 2026.

Other methods of identifying studies
Other studies will be identified by: *contacting authors or experts*, *looking through all the articles that cite the papers included in the review ("snowballing" or forward citation searching)*, *reference list checking (backward citation searching)*, *searching conference proceedings* and *searching dissertation and thesis databases*.

Additional information about identifying studies
Hand-searching Ghana Health Service website and repository, Ministry of Health Ghana publications, WHO AFRO Regional Office documents, West African Health Organisation (WAHO) reports, Ghana FELTP program documents, technical reports from development partners (CDC, USAID), and Joint External Evaluation reports for Ghana.

Link to search strategy
A full search strategy has been uploaded to PROSPERO. The PDF may be accessed through

this link https://www.crd.york.ac.uk/PROSPEROFILES/13a28f7f3e72f680a0ccfd37ecc2cad6.pdf.

Selection process

Studies will be screened independently by at least two people (or person/machine combination) with a process to resolve differences.

Other relevant information about searching and screening

All citations will be exported to EndNote reference management software and duplicates removed. Title and abstract screening followed by full-text review will be conducted independently by two reviewers using Covidence systematic review software.

Inter-rater reliability will be calculated using Cohen's kappa statistic (target ≥0.70). Disagreements will be resolved through discussion, with a third reviewer consulted if consensus cannot be reached.

No language restrictions will be applied during searching, but only studies in English and French will be included in final analysis due to resource constraints. For potentially eligible studies in other languages, titles and abstracts will be translated using Google Translate for initial screening.

Grey literature will be assessed for quality using the Authority, Accuracy, Coverage, Objectivity, Date, Significance (AACODS) checklist.

Study authors and Ghana Health Service officials will be contacted to identify unpublished studies, internal reports, and additional data from completed evaluations.

Search will be updated before final manuscript submission.

DATA COLLECTION PROCESS

Data extraction from published articles and reports

Data will be extracted by one person (or a machine) and checked by at least one other person (or machine).

Authors will not be contacted for further information.

Study risk of bias or quality assessment

Risk of bias will be assessed using:

Multiple quality assessment tools will be used depending on study design: JBI Critical Appraisal Checklist for cross-sectional studies, CASP (Critical Appraisal Skills Programme) Qualitative Checklist for qualitative studies, MMAT (Mixed Methods Appraisal Tool) for mixed-methods studies, AACODS checklist for grey literature, and Newcastle-Ottawa Scale adapted for non-randomized studies where applicable.

Data will be assessed independently by at least two people (or person/machine combination) with a process to resolve differences.

Additional information will be sought from study investigators if required information is unclear or unavailable in the study publications/reports.

Reporting bias assessment

Publication bias will be assessed through funnel plots and Egger's regression test if ≥10 studies are included in meta-analysis. We will search trial registries and contact Ghana Health Service to identify unpublished evaluations. Comparison of published versus grey literature findings will assess selective reporting.

Certainty assessment

Certainty of evidence will be assessed using an adapted GRADE (Grading of Recommendations Assessment, Development and Evaluation) approach suitable for complex public health interventions and observational studies.

Evidence will be rated across five domains: risk of bias (study quality), inconsistency (heterogeneity of results), indirectness (applicability to review question), imprecision (sample size and confidence intervals), and publication bias.

Given the nature of public health systems research, we will also consider: coherence (logical consistency of findings), adequacy of data (richness and quantity of evidence), and relevance to Ghana context.

Certainty will be classified as high, moderate, low, or very low for each main outcome. Two reviewers will independently assess certainty with discrepancies resolved through discussion.

For grey literature and implementation research, we will apply GRADE adaptations recognizing that randomized trials may not be feasible or appropriate for assessing health system preparedness.

Summary of Findings tables will present certainty ratings for main outcomes along with effect estimates, ensuring transparent presentation of evidence quality to inform policy recommendations.

OUTCOMES TO BE ANALYSED

Main outcomes

Primary Outcomes:

1. Overall Preparedness Level: Measured using standardized assessment scores (Joint External Evaluation scores, State Party Annual Reporting scores, IHR core capacity indicators) or composite preparedness indices. Time point: most recent assessment available.

2. Trained Workforce Capacity: Number and proportion of trained field epidemiologists (GFELTP graduates - advanced and frontline), biostatisticians, and disease surveillance officers per population or health facility. Competency assessment scores where available. Time point: current workforce status or post-training assessments.

3. Surveillance System Performance: Integrated Disease Surveillance and Response (IDSR)

indicators including completeness of reporting (percentage of expected reports received), timeliness (percentage of reports submitted within deadline), data quality scores, and sensitivity of outbreak detection. Time point: quarterly or annual surveillance performance data.

4. Outbreak Detection and Response Capacity: Time from disease onset to outbreak detection (detection timeliness), time from detection to response initiation, proportion of outbreaks investigated within 48 hours, completeness of outbreak investigation reports. Time point: analysis of outbreak events over study period.

5. Statistical Modeling and Analytical Capacity: Availability of biostatistical expertise (number of trained biostatisticians), use of statistical software, documented use of statistical models for decision-making, publications of epidemiological analyses. Time point: current capacity assessments.

Effect measures: Proportions, means, medians, scores on validated assessment tools, time-to-event measures, and trends over time where longitudinal data available.

Additional outcomes

Secondary Outcomes:

1. Infrastructure and Technology: Availability and functionality of public health laboratories with diagnostic capacity for priority diseases, information technology infrastructure (computers, internet connectivity), statistical software availability (Epi Info, R, STATA, SPSS), geographic information systems, electronic data capture systems. Measurement: inventory counts, functionality assessments.

2. Training Program Effectiveness: Ghana FELTP completion rates, post-training competency scores, trainee deployment to intended positions, retention in field epidemiology roles at 1, 3, and 5 years post-graduation, trainee project outputs (outbreak investigations, surveillance evaluations).

3. Data Management Systems: Implementation status and utilization of DHIMS2, electronic surveillance platforms, mobile-based reporting systems. Data quality metrics including completeness, accuracy, timeliness, consistency.

4. Laboratory Network Capacity: Number of laboratories with biosafety level 2 or 3 capacity, specimen transportation systems, laboratory confirmation rates for suspected outbreaks, laboratory result turnaround times.

5. Resource Allocation: Budget allocated to disease surveillance and outbreak response, availability of transportation for outbreak investigation, communication equipment, personal protective equipment stockpiles.

6. Policy and Governance: Existence of updated standard operating procedures for outbreak investigation, national preparedness plans, coordination mechanisms between health system levels, integration of statistical evidence into policy documents.

7. Geographic and Equity Considerations: Distribution of preparedness capacity across regions (urban vs rural, northern vs southern regions), equity in resource allocation, accessibility of

surveillance and response services.

8. Documented Performance: Number of published outbreak investigations, participation in international surveillance networks, contributions to regional epidemiological bulletins, evidence of south-south collaboration.

Time points vary by outcome but generally reflect most recent data available during study period (2010-2025).

Effect measures: Counts, proportions, scores, presence/absence of key elements, trends over time.

PLANNED DATA SYNTHESIS

Strategy for data synthesis

Narrative Synthesis:

A comprehensive narrative synthesis will be conducted for all included studies following guidance from the Cochrane Handbook and Popay et al. framework. Data will be organized thematically according to the five main domains: (1) infectious disease surveillance capacity, (2) outbreak investigation preparedness, (3) field epidemiology workforce, (4) statistical and biostatistical capacity, and (5) infrastructure and resources.

Studies will be tabulated with key characteristics (author, year, design, setting, sample, outcomes, findings, quality rating). Within each domain, findings will be synthesized descriptively, identifying patterns, relationships, and contradictions across studies. Textual descriptions will be supported by summary tables and figures.

Findings will be mapped against WHO IHR (2005) core capacity framework to identify specific gaps. Geographic distribution of capacity across Ghana's regions and variation by health system level (national, regional, district) will be analyzed.

Meta-Analysis:

Quantitative meta-analysis will be conducted if ≥3 comparable studies report similar outcomes with sufficient data. Random-effects models will be used to account for expected heterogeneity using the DerSimonian-Laird method. Analysis will be performed using Stata 17.0 or RevMan 5.4.

Potential meta-analyses include:

Pooled prevalence of trained field epidemiologists per population

IDSR performance indicators (completeness and timeliness) across regions/time periods

Training program completion rates

Outbreak detection times

Preparedness assessment scores

Effect measures: Risk ratios or odds ratios for dichotomous outcomes, mean differences or standardized mean differences for continuous outcomes, proportions for prevalence data. 95% confidence intervals will be calculated for all estimates.

Heterogeneity Assessment:

Statistical heterogeneity will be assessed using I^2 statistic and interpreted as: 0-40% (low), 30-60% (moderate), 50-90% (substantial), 75-100% (considerable). Chi-squared test (p<0.10 indicating significant heterogeneity) and Tau^2 will also be reported.

Subgroup Analyses:

If sufficient studies exist, subgroup analyses will examine:

Geographic region (Greater Accra, Ashanti, Northern regions, etc.)

Health system level (national, regional, district)

Time period (2010-2015, 2016-2020, 2021-2025)

Study design (observational vs. intervention studies)

Disease category (epidemic-prone, vaccine-preventable, emerging)

Sensitivity Analyses:

Sensitivity analyses will assess robustness of findings by:

Excluding studies with high risk of bias

Excluding grey literature

Using fixed-effect vs random-effects models

Excluding outlier studies

Publication Bias:

If ≥10 studies in meta-analysis, funnel plots will be generated and asymmetry assessed using Egger's regression test (p<0.10 significant).

Quality of Evidence:

GRADE approach will summarize certainty of evidence in Summary of Findings tables for main outcomes.

If meta-analysis is not feasible due to heterogeneity or insufficient studies, comprehensive narrative synthesis with vote counting based on direction of effect will be used, clearly noting limitations.

CURRENT REVIEW STAGE

Stage of the review at this submission

Review stage	Started	Completed
Pilot work	✓	✓
Formal searching/study identification	✓	✓
Screening search results against inclusion criteria		
Data extraction or receipt of IPD		
Risk of bias/quality assessment		
Data synthesis		

Review status

The review is currently planned or ongoing.

Publication of review results

Results of the review will be published in English.

REVIEW AFFILIATION, FUNDING AND PEER REVIEW

Review team members

Victor Luckyboy Dzramado (review guarantor and contact) ORCID: 0009-0007-1738-963X. Cape Coast Teaching Hospital. Ghana.

No conflict of interest declared.

Dr William Wilberforce Amoah. Kwame Nkrumah University of Sience and Technology. Ghana.

No conflict of interest declared.

Mr Samuel Antwi. University of Ghana Legon. Ghana.

No conflict of interest declared.

Miss Joana Edem Koto. Kintampo Health Research Institute. Ghana.

No conflict of interest declared.

Professor Doris Hagan. Kwame Nkrumah University of Science and Technology. Ghana.

No conflict of interest declared.

Named contact

Victor Luckyboy Dzramado (dzramadovictor@gmail.com). ORCID: 0009-0007-1738-963X. Cape Coast Teaching Hospital. Ghana.

Review affiliation

University of Cape Coast, Cape Coast, Ghana

Cape Coast Teaching Hospital

Funding source

Review has no funding and no agreed support from an academic institution and is done in authors' own time.

Peer review

The review protocol has undergone internal peer review by the academic supervisor (Dr. Obed U. Lasim) at the Department of Health Information Management, University of Cape Coast. The protocol was reviewed for methodological rigor, feasibility, and alignment with MPhil program requirements. Feedback was incorporated regarding search strategy comprehensiveness, inclusion/exclusion criteria clarity, and outcome selection. The protocol will undergo further peer review during journal submission process following PROSPERO registration.

ADDITIONAL INFORMATION

Review conflict of interest

Declared individual interests are recorded under team member details.. No additional interests are recorded for this review.

Medical Subject Headings

Biostatistics; Capacity Building; Classification; Communicable Diseases; Communication; Data Management; Disease Outbreaks; Electronics; Geographic Information Systems; Ghana; Health Services; Hospital Rapid Response Team; Humans; International Health Regulations; Public Health; Quality Improvement; Resource-Limited Settings; Sentinel Surveillance; Software; Systematic Reviews as Topic; Technology; Workforce; World Health Organization

SIMILAR REVIEWS

Check for similar records already in PROSPERO

PROSPERO identified a number of existing PROSPERO records that were similar to this one (last check made on 3 February 2026). These are shown below along with the reasons given by that the review team for the reviews being different and/or proceeding.

- A Decade of IDSR in Africa (2012–2024): Systematic Review of Implementation and Performance [published 2 November 2025] [CRD420251176296]. The review was judged **not to be similar**
- Analysis of the containment capacity and strategies to prevent the spread of filoviral outbreaks in Health Systems. [published 3 October 2024] [CRD42024593222]. The review was judged **not to be similar**
- Capacity for Infectious Disease Diagnostics in African Health Systems: A Systematic Review of Test Availability, Accessibility and Barriers [published 16 October 2025]

[CRD420251164504]. The review was acknowledged as **similar** but the authors opted to continue because *there are differences in population*, *there are differences in intervention or comparator*, *the review looks at additional or different outcomes*, *the review will be more up to date*

- Strengthen Health System Resilience to Emerging Infectious Diseases: Lessons from Recent Outbreaks [published 3 June 2025] [CRD420251027821]. The review was acknowledged as **similar** but the authors opted to continue because *there are differences in population*, *there are differences in intervention or comparator*, *the review looks at additional or different outcomes*, *the review will be more up to date*
- Towards sustainable implementation of digital tools for communicable disease surveillance and outbreak response in Africa: a systematic review of project costs, cost distribution, and funding practices [published 4 February 2022] [CRD42022300849]. The review was acknowledged as **similar** but the authors opted to continue because *there are differences in population*, *there are differences in intervention or comparator*, *the review looks at additional or different outcomes*, *the review will be more up to date*
- Comparing emerging infectious disease surveillance and early warning systems: a systematic review of multi-country practices after COVID-19 [published 7 January 2026] [CRD420251275385]. The review was acknowledged as **similar** but the authors opted to continue because *there are differences in population*, *there are differences in intervention or comparator*, *the review looks at additional or different outcomes*, *the review will be more up to date*
- Advancing Public Health Surveillance with Artificial intelligence: A Systematic Review of Real-Time Data Analytics and Disease Prediction [published 8 April 2025] [CRD420251013879]. The review was acknowledged as **similar** but the authors opted to continue because *there are differences in population*, *there are differences in intervention or comparator*, *the review looks at additional or different outcomes*, *the review will be more up to date*

PROSPERO version history

- Version 1.0, published 03 Feb 2026

Disclaimer

The content of this record displays the information provided by the review team. PROSPERO does not peer review registration records or endorse their content.

PROSPERO accepts and posts the information provided in good faith; responsibility for record content rests with the review team. The guarantor for this record has affirmed that the information provided is truthful and that they understand that deliberate provision of inaccurate information may be construed as scientific misconduct.

PROSPERO does not accept any liability for the content provided in this record or for its use. Readers use the information provided in this record at their own risk.

Any enquiries about the record should be referred to the named review contact
